# First report of the soft tick *Ornithodoros mimon* (Ixodida: Argasidae) in Alagoas State, Northeastern Brazil

**DOI:** 10.1590/S1984-29612024056

**Published:** 2024-09-16

**Authors:** Epitácio Correia de Farias, Ana Cecília Pires de Azevedo Lopes, Glauber Meneses Barboza Oliveira, Rafael Felipe da Costa Vieira, Marcelo Bahia Labruna, Jonatas Campos de Almeida

**Affiliations:** 1 Centro de Engenharia e Ciências Agrárias, Universidade Federal de Alagoas – UFAL, Viçosa, AL, Brasil; 2 Centro de Triagem de Animais Silvestres, Instituto do Meio Ambiente, Maceió, AL, Brasil; 3 Departamento de Medicina Veterinária Preventiva e Saúde Animal, Faculdade de Medicina Veterinária e Zootecnia, Universidade de São Paulo – USP, São Paulo, SP, Brasil; 4 Department of Epidemiology and Community Health, College of Health and Human Services, The University of North Carolina at Charlotte, Charlotte, NC, USA; 5 Center for Computational Intelligence to Predict Health and Environmental Risks, The University of North Carolina at Charlotte, Charlotte, NC, USA

**Keywords:** Argasid, opossum, Didelphis albiventris, Argasídeo, gambá, Didelphis albiventris

## Abstract

The Brazilian tick fauna currently comprises 77 valid species categorized into two families: Ixodidae (53 species) and Argasidae (24 species). In the state of Alagoas, only six Ixodid ticks have been reported to date, with no previous reports of ticks in the Argasidae family. Here, we assessed 33 White-eared Opossum (*Didelphis albiventris* Lund, 1840) rescued in the metropolitan region of Maceió and referred to the Wild Animal Screening Center (Cetas) in the city. Upon arrival, the animals were examined for ectoparasites within 24 hours. In total, 10/33 (30%) opossums were found to be infested by 26 larvae of the argasid tick *Ornithodoros mimon* Kohls, Clifford & Jones, 1969. Morphological identification of ticks was corroborated by generating partial sequences of the mitochondrial 16S rRNA gene from three tick specimens. This study marks the first report of an argasid tick in the state of Alagoas. Future studies should investigate whether populations of both *O. mimon* ticks and their host, *D. albiventris*, in the state of Alagoas carry potential zoonotic agents capable of causing tick-borne diseases.

## Resumo

A fauna de carrapatos do Brasil compreende, atualmente, 77 espécies válidas, categorizadas em duas famílias: Ixodidae (53 espécies) e Argasidae (24 espécies). No estado de Alagoas, apenas seis carrapatos ixodídeos foram registrados até o momento, sem nenhum relato anterior de argasídeos. Neste estudo, 33 gambás-de-orelha-branca (*Didelphis albiventris* Lund, 1840), resgatados na região metropolitana de Maceió, foram encaminhados ao Centro de Triagem de Animais Silvestres (Cetas) da cidade. Ao chegarem, os animais foram submetidos a exames de ectoparasitas em um prazo de 24 horas. Do total, 10/33 (30%) deles foram encontrados infestados por 26 larvas do carrapato argasídeo *Ornithodoros mimon* Kohls, Clifford & Jones, 1969. A identificação morfológica dos carrapatos foi corroborada pela obtenção de sequências parciais do gene mitocondrial 16S rRNA de três espécimes deles. Este estudo marca o primeiro relato de um carrapato argasídeo no estado de Alagoas. Estudos futuros devem investigar se as populações de ambos os carrapatos, *O. mimon* e seu hospedeiro, *D. albiventris*, no estado de Alagoas, carregam potenciais agentes zoonóticos capazes de causar doenças transmitidas por carrapatos.

Within the global arthropod fauna, ticks are known for transmitting the greatest variety of microorganisms, including viruses, bacteria, protozoa, and filarial nematodes (Sonenshine & Roe, 2014). For this reason, knowledge of the tick fauna in each region is essential for local determinations of the risks of tick-borne diseases and for planning actions to diagnose, prevent, and control these diseases in the context of One Health. In Brazil, there are currently 77 valid tick species divided into two families: Ixodidae (53 species) and Argasidae (24 species) (Labruna et al., 2024). Although studies on ticks and tick-borne diseases have made undeniable advances during recent decades in Brazil (reviewed by Barros-Battesti et al., 2024), these studies have been unevenly focused within the country's large geographical area. A clear example is the state of Alagoas, where, until last year, only the following four tick species have been reported: *Amblyomma varium* Koch, 1844; *Ixodes amarali* Fonseca, 1935; *Ixodes loricatus* Neumann, 1899; and *Rhipicephalus microplus* (Canestrini, 1888) (Aragão, 1936; Barros-Battesti & Knysak, 1999; Marques et al., 2002). A new study in 2023 reported the species *Amblyomma sculptum* Berlese, 1888, and *Dermacentor nitens* Neumann, 1897, for the first time in the state (Gama et al., 2023), increasing Alagoas' local tick fauna to six species, all from Ixodidae and representing only 7.8% of the Brazilian tick fauna. Notably, Alagoas has not had any reports of ticks from the Argasidae family. This study reports for the first time a species of argasid tick in Alagoas by studying tick infestations on opossums rescued in a wildlife facility.

During November and December of 2022, 33 individuals of the white-eared opossum *Didelphis albiventris* Lund, 1840 (Didelphimorphia: Didelphidae), were examined for ectoparasites. These animals were rescued in the metropolitan region of Maceió (09°39’S 35°43’W) and transported to the Wild Animal Screening Center (Cetas) of Maceió city. Upon arrival, the animals were examined for the presence of ectoparasites within 24 hours. When encountered, ticks were placed in plastic vials containing 70% ethanol and subsequently sent to the laboratory for taxonomic identification following the morphological methods outlined by Kohls et al. (1969) for argasid larvae. Attempts to confirm the morphological identification by molecular methods were performed with eight ticks, which were submitted to amplification via polymerase chain reaction (PCR) of a 460-bp partial sequence of the tick mitochondrial 16S rRNA gene following the protocol reported by Mangold et al. (1998). PCR products were purified and sequenced with a Big Dye Terminator Cycle Sequencing Kit (Applied Biosystems, Foster City, CA, USA) in an automatic sequencer (model ABI 3500 Genetic Analyzer; Applied Biosystems) according to the manufacturer’s protocol. The generated sequences were subjected to BLAST analysis (Altschul et al., 1990) to infer the closest identities to tick DNA sequences available in the GenBank® database.

**Figure 1:**
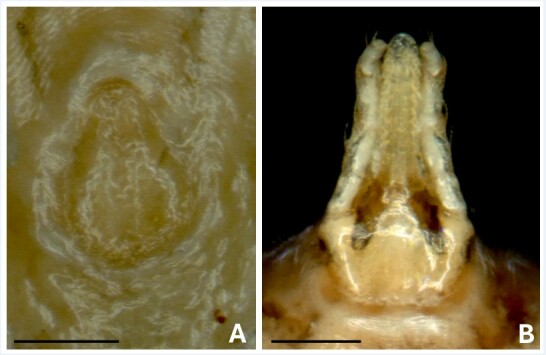
*Ornithodoros mimon* larva collected from White-eared Opossum in the present study. Dorsal plate (A) and ventral gnathosoma (B). Bars: 100 µl.

Among the 33 examined white-eared opossums, 10 (30%) were infested by ticks, which were identified as 26 larvae of the argasid tick *Ornithodoros mimon* Kohls, Clifford & Jones, 1969, with a mean intensity of infestation of 2.6 larvae per infested animal (range: 1-6). Among the eight *O. mimon* larvae processed by molecular analysis, PCR amplification was successful for only three larvae, which yielded the same 16S rRNA haplotype (deposited in GenBank under accession number PP731806). By BLAST analysis, this haplotype was 99.8% identical (422/423 nucleotides) to Brazilian haplotypes of *O. mimon* from the states of Mato Grosso (ON800866) and Pernambuco (KC677676). The remaining 18 larvae were deposited in the tick collection “Coleção Nacional de Carrapatos Danilo Gonçalves Saraiva” (CNC) under the accession number CNC-4754. Morphological identification of *O. mimon* larvae relied on the presence of 14 pairs of dorsal setae, 8 pairs of ventral setae, pear-shaped dorsal plate (Figure 1A), and hypostome apically blunt (Figure 1B).

This study provides the first report of an argasid tick from the state of Alagoas. Previous studies have reported *O. mimon* in 10 other Brazilian states: Goiás, Minas Gerais, Mato Grosso do Sul, Pernambuco, Rio Grande do Norte, Rio de Janeiro (Labruna et al., 2014; Lourenço et al., 2023), Mato Grosso (Muñoz-Leal et al., 2021), Maranhão (Costa et al., 2020), Ceará (Jorge et al., 2022), and São Paulo (Oliveira et al., 2023). This distribution includes highly humid areas of the Amazon and Atlantic Forest biomes, areas of the savannah-like Cerrado biome, and areas dominated by semi-arid climate in the Caatinga biome (Ab’Sáber, 2003). Indeed, this apparent ecological plasticity of *O. mimon* is facilitated by its nidicolous lifestyle, typical of the argasid ticks (Vial, 2009). The present record in Alagoas is within the Atlantic Forest of Northeastern Brazil, where *O. mimon* was previously reported in the neighboring state of Pernambuco (Labruna et al., 2014). If on the one hand the present report does not present an ecological novelty, on the other it denotes a huge gap in studies on ticks in Brazil, since the biggest surprise is the fact that this tick has remained unknown for the state of Alagoas until now. This scenario portrays the disparity in current knowledge of ticks in a large country like Brazil.

While the original description of *O. mimon* referred to this species as a parasite of bats in Bolivia and Uruguay (Kohls et al., 1969), in Brazil, most of the host records of *O. mimon* have been on marsupials, especially the White-eared Opossum (Lopes et al., 2018; Sponchiado et al., 2015; Silva et al., 2017; Barbieri et al., 2019). Furthermore, among the Brazilian argasid fauna, *O. mimon* stands out as one of the species with the greatest number of human bite records (Nogueira et al., 2022). In fact, there are several records of *O. mimon* colonizing the roofs of human homes, leading to ticks attacking humans during the night. In these cases, roofs were also inhabited by bats and/or opossums, which likely sustained the *O. mimon* populations within the homes (Labruna et al., 2014; Dantas-Torres et al., 2022; Oliveira et al., 2023).

This study is of great public health relevance for Alagoas. In addition to *O. mimon* being a tick known to bite humans, it is considered a potential vector for a new *Borrelia* of the relapsing fever group, ‘*Candidatus* Borrelia mimona’, recently detected and isolated from white-eared opossums in the state of São Paulo (Weck et al., 2024). Furthermore, a new haplotype of *Rickettsia* from the spotted fever group was recently identified in *O. mimon* ticks in Rio de Janeiro (Dantas-Torres et al., 2022). Future studies should investigate whether populations of both *O. mimon* ticks and their host, *D. albiventris*, in the state of Alagoas carry potential zoonotic agents capable of causing tick-borne diseases.
